# Cord blood presepsin as a predictor of early-onset neonatal sepsis in term and preterm newborns

**DOI:** 10.1186/s13052-023-01420-z

**Published:** 2023-03-21

**Authors:** Francesca Priolo, Luca Maggio, Simona Fattore, Marta Tedesco, Domenico Umberto De Rose, Alessandro Perri, Giorgia Prontera, Roberto Chioma, Annamaria Sbordone, Maria Letizia Patti, Giovanni Vento

**Affiliations:** 1 Department of Woman and Child Health and Public Health, Neonatology Unit, Fondazione Policlinico Universitario “A. Gemelli” IRCCS, Largo A. Gemelli 8, 00168 Rome, Italy; 2 Catholic University of the Sacred Heart, Rome, Italy; 3 Neonatal Intensive Care Unit, Maternal-Fetal Department, “S. Camillo-Forlanini” Hospital, Rome, Italy; 4 Department of Fetus-Newborn-Infant, Neonatal Intensive Care Unit, Medical and Surgical, “Bambino Gesù” Children’s Hospital IRCCS, Rome, Italy

**Keywords:** Presepsin, Neonatal sepsis, Newborn, Antibiotic therapy, Biomarker, Cord blood

## Abstract

**Background:**

To date, no studies on presepsin values in cord blood of term infants with risk factors for early-onset sepsis (EOS) are available, whereas only one study reported presepsin values in cord blood of preterm infants at risk. In this study, we investigated the presepsin values in cord blood of term and preterm infants with documented risk factors for EOS.

**Methods:**

In this single-center prospective pilot study, we enrolled neonates presenting with documented risk factors for EOS. P-SEP levels were assessed in a blood sample collected from the clamped umbilical cord after the delivery in 93 neonates, using a point-of-care device. The primary outcome of our study was to evaluate the role of cord blood P-SEP in predicting clinical EOS in term and preterm infants.

**Results:**

During the study period, we enrolled 93 neonates with risk factors for EOS with a gestational age ranging between 24.6 and 41.6 weeks (median 38.0). The median P-SEP value in all infants was 491 pg/ml (IQR 377 – 729). Median cord P-SEP values were significantly higher in infants with clinical sepsis (909 pg/ml, IQR 586 – 1307) rather than in infants without (467 pg/ml, IQR 369 – 635) (*p* = 0.010). We found a statistically significant correlation between cord P-SEP value at birth and the later diagnosis of clinical sepsis (Kendall's τ coefficient 0.222, *p* = 0.002). We identified the maximum Youden’s Index (best cut-off point) at 579 pg/ml, corresponding to a sensitivity of 87.5% and a specificity of 71.8% in predicting clinical sepsis.

**Conclusions:**

Maximum Youden’s index was 579 pg/ml for clinical EOS using cord P-SEP values. This could be the starting point to realize multicenter studies, confirming the feasibility of dosing P-SEP in cord blood of infants with risk factors of EOS to discriminate those who could develop clinical sepsis and spare the inappropriate use of antibiotics.

## Background

The term “Early-onset Sepsis” (EOS) refers to sepsis that usually occurs in the first 72 h of life, caused by microorganisms transmitted vertically from the mother to the newborn [1]. The incidence of EOS is estimated to be 0.5–2 per thousand live births [2]. The organisms mainly involved in EOS colonize the birth canal, such as group B streptococcus (GBS) and *Escherichia coli*. They are responsible for 70% of EOS [1]. EOS diagnosis represents a challenge for pediatricians: clinical symptoms are not specific, and there is no consensus about the best sepsis definitions and biomarkers to use [2, 3]. The administration of the first dose of antibiotics within the golden hour in infants with sepsis is critical because any delay can increase mortality [4].

Among the different existing biomarkers, none showed satisfactory sensitivity and specificity values in EOS diagnosis [5]. Currently, the most used biomarkers in clinical routine are C-reactive protein (CRP) and procalcitonin (PCT). However, serum levels of CRP and PCT show a physiological increase during the first 48–72 h of life and are influenced by several maternal and fetal pro-inflammatory conditions, other than infections [6, 7].

Presepsin (P-SEP) is the N-terminal fragment of soluble CD14 subtype (sCD14-ST): it is released in the bloodstream by monocytes and macrophages, in response to the contact of the immune system with a pathogen. P-SEP seems to be a new, promising biomarker for the early diagnosis of sepsis in neonates as it is not modified by perinatal confounding inflammatory factors [8]. A recent meta-analysis, including 12 studies and 828 newborns of any gestational age with a diagnosis of EOS, revealed that P-SEP had a pooled sensitivity and specificity of 0.93 (95% CI, 0.86–0.95) and 0.91 (95% CI, 0.85–0.95), respectively [9].

The real challenge remains the identification of newborns at high risk for EOS before clinical symptoms occur and identifying those who need prophylactic antibiotic therapy. Seliem and Sultan previously measured P-SEP in cord blood of preterm infants with premature rupture of membranes, finding that umbilical P-SEP is a good predictor of EOS and may help to reduce the misuse of antibiotics [10]. No studies are available on the measurement of P-SEP in cord blood of term infants. Our aim was to evaluate the role of cord blood P-SEP in predicting clinical EOS in term and preterm infants.

## Materials and methods

### Study design

In this prospective pilot study, we considered for enrolment all neonates with risk factors for EOS, assessed at Fondazione Policlinico Universitario “A. Gemelli” IRCCS (Rome, Italy) from June 2019 to February 2021. We considered eligible for inclusion in the study every infant presenting with at least one of the following risk factors: (1) maternal chorioamnionitis (defined as maternal intrapartum temperature ≥ 39,0 °C, or maternal intrapartum temperature of 38,0–39,0 °C for more than 30 min and at least one of the following signs: fetal tachycardia, purulent amniotic fluid, maternal leucocytosis > 15,000/mm^3^) or (2) inadequate intrapartum antibiotic prophylaxis or (3) lack of antibiotic prophylaxis when this is indicated by the current CDC guidelines [11].

Exclusion criteria were: (1) congenital TORCH infections; (2) congenital anomalies or hydrops fetalis; 3) absence of written informed consent to participate from a legal guardian.

EOS was defined within the first 72 h of life either by the presence of positive blood culture (BC) or as clinical sepsis (in the absence of a positive BC), according to definite criteria (Table 1) [12]. Blood cultures were taken for all infants admitted to the Neonatal Intensive Care Unit (NICU) or Neonatal Intermediate−Care Unit.

**Table 1:** Our internal protocol with clinical signs and red flags of sepsis. Clinical sepsis, with no positive blood culture, was diagnosed in presence of at least two clinical signs or at least one red flag sign

Clinical signs	Abnormal reactivity Abnormal tone Feeding intolerance (vomit, aspirates, abdominal distension) Bradycardia or tachycardia Central cyanosis or low SpO2 Jaundice within the first 24 h of life Apnoea Hypothermia (< 36 °C) or hyperthermia (> 38 °C) Thrombocytopenia or coagulopathy (INR > 2) with no obvious cause Persisting oliguria (more than the first 24 h of life) Hypoglycemia or hyperglycemia (beyond an adequate glucose intake) Metabolic acidosis (BE ≥ -10 mmol/L) Localized infectious signs (skin, eyes)
Red flags	Respiratory distress whose onset is at 4 or more hours of life Seizures Mechanical ventilation in term infants Shock

The probability of EOS based on maternal risk factors and the infant’s clinical presentation was assessed using an interactive calculator (available at: https://neonatalsepsiscalculator.kaiserpermanente.org/) [13].

At birth, for any eligible infant, a venous blood sample was collected from clamped cord before placental expulsion. EDTA coated syringes were used, and any sample was analyzed to get the plasmatic presepsin concentrations through PATHFAST® presepsin, a chemiluminescent enzyme immunoassay, using the PATHFAST® point-of-care analyzer [8].

We performed a blinded analysis so that for the clinical and therapeutic management of the newborns the current neonatal guidelines were followed, regardless of the P-SEP value.

We obtained clinical data of mothers and neonates from medical electronic records, collecting variables such as gestational age (GA), birthweight, the rate of weight appropriate for GA (AGA) / small for GA (SGA) / large for GA (LGA), maternal age, delivery mode, Apgar score at 1^st^ and 5^th^ minute, in-hospital mortality, length of stay, CRP values at 6 and 12 h, score at Kaiser Permanente Neonatal Sepsis calculator, adverse neonatal outcomes (including neonatal fever, feeding intolerance, tone anomalies, hypo/hyperglycemia, respiratory distress, need of mechanical ventilation, brady/tachycardia, jaundice, thrombocytopenia, abdominal distension, cyanosis, seizures, clinical EOS), maternal vaginal swabs, maternal urine culture, intrapartum antibiotic prophylaxis, highest maternal body temperature, length of the premature rupture of membranes.

The primary outcome of our study was to evaluate the role of cord blood P-SEP in predicting clinical EOS in term and preterm infants. We also evaluated if P-SEP levels were correlated with GA and days of antibiotic therapy.

### Statistical analysis and ethical issues

Data were analyzed using Statistical Package for Social Science 25.0 version (SPSS, Inc, Chicago, IL, USA). Categorical variables are presented as numbers and percentages, while continuous variables are presented as the mean and standard deviation (if they were normally distributed) or as the median and interquartile range (if normality could not be accepted). Data distribution was evaluated by the Shapiro–Wilk test.

P-SEP values were set as independent variables and GA, EOS, and length of antibiotic therapy as dependent variables in linear regression models, using Kendall's Tau and Spearman's Rank Correlation Coefficient as appropriate.

Furthermore, we divided our population into two groups according to P-SEP reference values for neonates [14]: we compared infants with P-SEP higher than 50^th^ centile versus those with a value lower than 50^th^ centile (604 pg/ml for term babies and 620 pg/ml for preterm ones, respectively), higher than 75^th^ centile versus those with a value lower than 75^th^ centile (791 pg/ml for term babies and 864 pg/ml for preterm ones, respectively), higher than 90^th^ centile versus those with a value lower than 90^th^ centile (1000 pg/ml for term babies and 1060 pg/ml for preterm ones, respectively).

Differences among groups were assessed using Fisher’s exact test or Mann–Whitney test, as appropriate. Sensitivity, specificity, positive predictive value, and negative predictive value of P-SEP in the diagnosis of clinical EOS were calculated.

Receiver operating characteristic (ROC) analysis was computed, and the area under the ROC curve (AUC) and Youden’s index were used to evaluate the ability of cord blood P-SEP to predict clinical EOS in neonates.

A *p*-value < 0.05 was considered significant: two-sided *p*-values are reported.

The study was approved by the Institutional Ethics Committee of the Fondazione Policlinico Universitario “A. Gemelli” IRCCS, Rome, Italy (Prot.36099/19-ID 2751). Sample size was not determined a priori. Written informed consent was obtained from newborns’ parents before inclusion in the study.

## Results

During the study period, P-SEP levels were assessed in a blood sample collected from the clamped umbilical cord after the delivery in 93 neonates with risk factors for EOS, with a GA ranging between 24.6 and 41.6 weeks (median 38.0). Twenty-nine neonates were born preterm (31.2%) and sixty-four were term infants (68.8%). Fifty-six neonates (60.2%) were vaginally delivered and thirty-seven (39.8%) by caesarean section. The characteristics of the study population were reported in Table 2.

**Table 2:** Characteristics of the study population. Continuous variables are expressed as median (25°-75° percentile), and categorical variables are expressed as numbers (percentage)

	***N* = 93**
Gestational age (weeks)	38.0 (36.0 – 40.0)
Preterm (< 37 weeks)	29 (31.2%)
Born before 34 weeks	12 (12.9%)
Late preterm (34–36 weeks)	17 (18.3%)
Birthweight (grams)	3095 (2615 – 3500)
SGA infants	4 (4.3%)
LGA infants	7 (7.5%)
1-min Apgar score	9 (8–9)
5-min Apgar score	10 (9–10)
Males	52 (55.9%)
Mode of delivery	
Non-operative vaginal delivery	49 (52.7%)
Vacuum-assisted vaginal delivery	8 (8.6%)
Cesarean section	36 (38.7%)
Maternal vaginal/rectal swabs	
Negative	27 (29.0%)
Unknown/not performed	34 (36.6%)
Positive for GBS	28 (30.1%)
Positive for E. coli	4 (4.3%)
Maternal urine culture	
Negative	15 (16.1%)
Unknown/not performed	64 (68.8%)
Positive for GBS	6 (6.5%)
Positive for E. coli	8 (8.6%)
Highest maternal antepartum temperature (°C)	36.6 (36.4 – 38.0)
Premature rupture of membranes (hours)	8 (1.0—20.0)
Type of intrapartum antibiotics	
Broad spectrum antibiotics > 4 h prior to birth	14 (15.1%)
Broad spectrum antibiotics 2–3.9 h prior to birth	17 (18.3%)
GBS specific antibiotics > 2 h prior to birth	15 (16.1%)
No antibiotics or any antibiotics < 2 h prior to birth	47 (50.5%)

Sixteen neonates (17.2%) required admission to the NICU and twenty neonates (21.5%) in Neonatal Intermediate-Care Unit. Sixty-two neonates remained with their own mothers in our Rooming-in ward (66.7%). Only one preterm infant died (1.1%).

The median P-SEP value was 491 pg/ml (IQR 377 – 729) in umbilical cord blood samples collected after the delivery.

Cord P-SEP values were unrelated to GA (Spearman's ρ coefficient -0.026, *p* = 0.804). Indeed, we found no significant differences in cord P-SEP values between term infants (median: 506 pg/ml; IQR 367—736) and preterm infants (median: 459 pg/ml; IQR 406 – 607) (*p* = 0.992). Similarly, we found no significant differences in cord P-SEP values between infants born ≤ 32 weeks GA (median: 502 pg/ml; IQR 411 – 1141) and infants born after 32 weeks GA (median: 491 pg/ml; IQR 376 – 646) (*p* = 0.535).

Twenty-four infants (25.8%) received antibiotics: antibiotics were prescribed in all preterm infants < 34 weeks GA, whereas in late preterm and term infants a mixed strategy was used (basing the choice on a risk factors approach and sepsis calculator score).

Clinical EOS was observed in 8/93 infants (8.6%); in the other cases, antibiotics were stopped before 72 h of life in the absence of positive cultures and symptoms. Blood culture was positive in 3/36 cases (8.3%): we detected a case of group B streptococcal (GBS) infection, a case of *Escherichia coli* infection, and a case of *Staphylococcus epidermidis* infection (labeled as an infection because of clinical symptoms).

Lumbar puncture was performed in 6/8 neonates with clinical sepsis and liquor culture resulted negative in all cases.

The median risk assessed at birth in infants born > 34 weeks GA by the sepsis calculator was in general 0.08 per 1000 births (IQR 0.02—0.40), whereas after the clinical exam was 0.06 per 1000 births (IQR 0.02 – 0.24). The general risk assessed by the sepsis calculator was not correlated with later diagnosis of clinical sepsis (τ -0.120, *p* = 0.111) and positive blood culture (τ -0.010, *p* = 0.880), whereas we confirm that the risk assessed after the clinical exam was significantly related to later diagnosis of clinical sepsis (τ 0.273, *p* = 0.000) and to positive blood culture (τ 0.185, *p* = 0.015).

Median cord P-SEP values were significantly higher in infants with clinical sepsis (909 pg/ml, IQR 586 – 1307) rather than in infants without (467 pg/ml, IQR 369 – 635) (*p* = 0.010).

We found a statistically significant correlation between cord P-SEP value at birth and the later diagnosis of clinical sepsis (τ 0.222, *p* = 0.002) and positive blood culture (τ 0.181, *p* = 0.011), whereas P-SEP values seemed to be not related to the length of antibiotic therapy (ρ 0.144, *p* = 0.167).

Furthermore, considering reference ranges of P-SEP in neonates, we found a significantly greater incidence of clinical sepsis in infants with cord P-SEP higher than the 50^th^ centile (5/27 vs 3/66, *p* = 0.043), higher than the 75^th^ centile (4/75 vs 4/18, *p* = 0.043), higher than the 90^th^ centile (4/79 vs 4/14, *p* = 0.016).

Table 3 reports the sensitivity, specificity, positive predictive value, negative predictive value, and accuracy of cord P-SEP in predicting clinical sepsis when the 50^th^ centile, 75^th^ centile, and 90^th^ centile are used.

**Table 3:** Sensitivity, specificity, positive predictive value, negative predictive value, and accuracy in predicting clinical sepsis when 50th centile, 75th centile, and 90th centile are used

	***Cord*** ***P-SEP value higher than 50***^***th***^*** centile***	***95% CI***	***Cord*** ***P-SEP value higher than 75***^***th***^*** centile***	***95% CI***	***Cord*** ***P-SEP value higher than 90***^***th***^*** centile***	***95% CI***
Sensitivity	62.5%	24.5–91.5%	50.0%	15.7–84.3%	50.0%	15.7–84.3%
Specificity	74.1%	63.5–83.0%	83.5%	73.9–90.7%	88.2%	79.4–94.2%
Positive Predictive Value	18.5%	10.6–30.3%	22.2%	11.0–39.9%	28.6%	13.9–49.7%
Negative Predictive Value	95.5%	89.5–98.1%	94.7%	89.8–97.3%	94.9%	90.3–97.4%
Accuracy	73.1%	62.9–81.8%	80.7%	71.2–88.1%	85.0%	76.0–91.5%

**Fig. 1:**
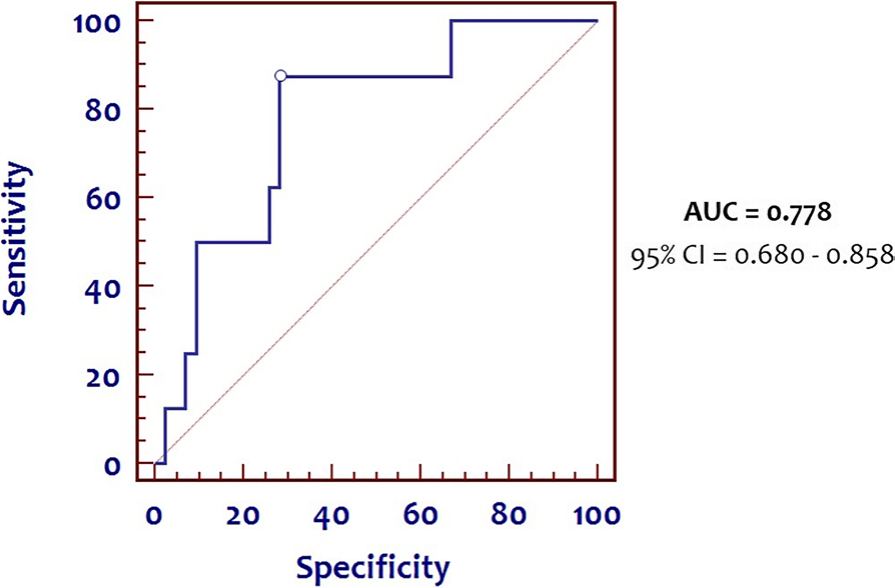
ROC curve of cord P-SEP values for clinical EOS in all infants

**Fig. 2:**
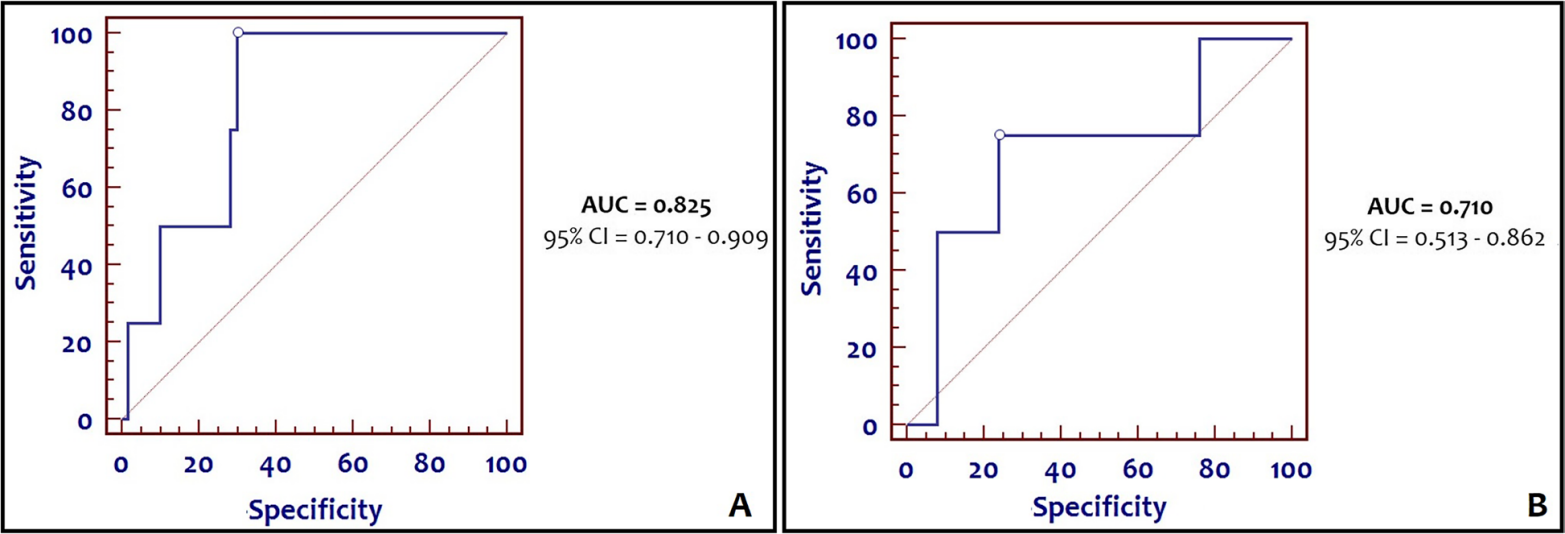
ROC curve of cord P-SEP values for clinical EOS in term infants (**A**) and in preterm infants (**B**)

Using the 50^th^ centile cut-off, we also created a ROC curve with an area under the curve (AUC) of 0.778 (95% CI = 0.680 – 0.858). We identified the maximum Youden’s Index (best cut-off point) at 579 pg/ml, corresponding to a sensitivity of 87.5% and a specificity of 71.8% (Fig**. ****1**). When P-SEP values of term infants and preterm infants were analyzed separately, we identified for term infants an AUC of 0.825 (95% CI = 0.710 – 0.909), with the maximum Youden’s index at 579 pg/ml, corresponding to a sensitivity of 100% and a specificity of 70% (Fig. **2****-**A). Conversely, we found for preterm infants an AUC of 0.710 (95% CI = 0.513 – 0.862), with the maximum Youden’s index at 544 pg/ml, corresponding to a sensitivity of 75% and a specificity of 76% (Fig. **2****-**B).

## Discussion

In this study, we measured P-SEP values in cord blood of term and preterm infants with risk factors for EOS, describing a significant correlation between cord presepsin and clinical early-onset sepsis.

Previously, Seliem and Sultan investigated whether presepsin levels in umbilical cord blood can be used as a predictor of EOS in preterm labor with premature rupture of membranes (PROM). They included only preterm infants born between 24 and 36 weeks of gestation, finding a higher P-SEP in cases with EOS (2231 pg/ml) than in those without (275 pg/ml) [10]. We confirm this trend, with higher P-SEP values in infants with clinical sepsis (909 pg/ml) rather than in infants without (467 pg/ml), but we included not only preterm neonates but also those term-born.

Another difference between our study and their one was the method to measure presepsin blood levels: they stored centrifuged samples (at 1000 × g for 15 min) at -20 °C until analysis and then measured P-SEP values using enzyme-linked immunosorbent assay (ELISA; Abbexa Ltd., Cambridge, UK). Instead, a step forward in the plasmatic measure of P-SEP is represented by a novel, highly sensitive and fully automated method, based on the chemiluminescence (CLEIA) method, providing results in 17 min in six samples simultaneously by a Point-of-Care Testing (POCT) instrument [8, 15]. In our study, we immediately analyzed cord blood samples using this new method although ELISA. The feasibility of this point-of-care method in measuring P-SEP values in EOS has been tested in different NICUs [9, 16–20]: this new method could be considered in the panoply of EOS risk assessment strategies used within neonatal units.

Despite a gradual improvement in trends, neonatal sepsis continues to be a major cause of morbidity and mortality, especially in the VLBW (Very Low Birth Weight) population, with an incidence of 1–4 / 1000 live births [21]. Thus, the early diagnosis of neonatal sepsis is essential due to the rapid evolution of the clinical situation. An early, sensitive and specific laboratory test would be helpful to guide clinicians in deciding whether or not to start antibiotics, avoiding unnecessary treatment, considering that more than 75% of VLBW infants receive prophylactic antibiotics because of the presence of EOS risk factors [22]. The use of antibiotics is not free from drug-related risks, but also risks related to the need for venous access, as well as the discussed risk of necrotizing enterocolitis [23]. Furthermore, exposure to antibiotics via maternal intrapartum antibiotic administration and neonatal administration after birth both alter the composition of the newborn's microbiome through infancy [22].

Currently, the gold standard for the diagnosis of sepsis is blood culture, although EOS is culture-confirmed in only about 1% of VLBW newborns: this rate was 20 times higher than that found in neonates born with birth weights higher than 2500 g according to Stoll’s findings [24]. Furthermore, response times require at least 48–72 h, and the percentage of false negatives related, in most cases, to intrapartum maternal antibiotic therapy, or the cases of false positives associated with contamination of the sample at the time of collection, is not negligible. The consequence of all this is that newborns with risk factors or in the presence of clinical suspicion are all treated with antibiotic therapy without any distinction.

Presepsin has now been widely tested as a marker of sepsis in term and preterm infants [8]. The availability of reference ranges of P-SEP values in the blood of term and preterm neonates has led to greater use of this marker in clinical routine. Pugni et al. found a median value of 603.5 pg/mL in the blood of uninfected term infants, whereas a median value of 620 pg/mL in that of uninfected preterm infants. The reference ranges of presepsin they determined were much higher than those seen in healthy adults, in whom a cut-off value of 600 ng/L has been reported for the discrimination of bacterial sepsis with a sensitivity and specificity of 87.8% and 81.4%, respectively [25]. Among the eventual causes, there is the activation of the innate immune system after birth, which occurs as a result of the shift from a usually sterile intrauterine environment to a world rich in foreign antigens. Furthermore, the newborn’s skin and stomach are rapidly colonized with microbial flora after birth, providing continual stimulation to the innate immune system [26].

Comparing cord P-SEP values of our infants to reference ranges of blood P-SEP values, we found that infants with clinical EOS had at birth higher values (greater than 75^th^ centile), while infants who did not develop clinical sepsis had initially lower values (lower than 50^th^ centile).

An interesting study has recently shown how high P-SEP values early correlate with sepsis’ onset, thus making it possible to identify newborns at higher risk, intervening before the presentation of clinical symptoms, and reducing the doses of antibiotics in lower-risk infants [20].

In recent years, evidence has emerged about using cord blood as a possible diagnostic tool for early sepsis. The measurement of acute phase proteins and cytokines such as CRP, PCT, interleukin-6 (IL-6), interleukin-8 (IL-8), tumor necrosis factor α (TNFα), and interleukin-1β (IL-1β) let us assess the fetal inflammatory response in utero [17], even if from currently available studies none of these markers would be able to confirm or exclude the diagnosis of EOS in the newborn [27].

Seliem and Sultan’s findings about cord presepsin in preterm infants who late developed EOS compared to their healthy peers were interesting: however, beyond the inclusion of only preterm infants, they did not report a cut-off of cord P-SEP to consider [10].

Conversely, our study included infants of all gestational ages with coded risk factors, as per CDC guidelines [11].

Moreover, both infants with positive blood cultures and infants with suspected sepsis were included in the definition of EOS, also in this case, in the presence of coded clinical signs, according to well-defined criteria. This choice derives from the small percentages of positive blood cultures in the neonatal population and from the fact that negative blood cultures do not allow excluding EOS in the presence of a compatible clinical picture. According to this, the incidence of clinical sepsis in our population was 8.6% (8/93 cases), while only in 3/93 cases sepsis was culture-proven (3.2%). This is a major limitation of our study because a discrete proportion of neonates might be ill because of conditions different from sepsis, leading to an overestimation of presepsin accuracy. Therefore, considering the low incidence of culture-proven early-onset sepsis in real life, beyond risk factors, a multicentric study about the presepsin values in cord blood including only infants with culture-proven EOS should be conducted.

The use of cord blood for the dosage of presepsin made it possible to avoid invasive procedures on the newborn (venipuncture, withdrawal from the heel). Furthermore, the point-of-care reading method allows for almost immediate results and seems to be feasible to use in neonatology units. The heterogeneity of the sample examined, including both premature and full-term infants, did not compromise the accuracy of the test studied, thus suggesting a possible transversal use. We identified an ideal cut-off point of 579 pg/ml (579 pg/ml for term infants and 544 pg/ml for preterm infants if considered separately) as an accurate screening method in neonates with risk factors for EOS, possibly avoiding administrating antibiotic prophylaxis in those with low cord P-SEP values.

The positive predictive value of presepsin in cord blood remained quite low with any cut-off, with the eventual risk of many false positive results. Similarly, correlation coefficients (Kendall’s tau), despite significant differences, were not high. Indeed, our findings are limited by the small sample size due to the single-center design and the low number of true positives. We could not assess its capability to discriminate between septic and non-septic patients among clinically ill newborns, because the low number of septic patients and the overlapping clinical picture with other disorders. Therefore, further multicentric studies are needed to confirm our findings. However, the identified value of presepsin in cord blood does not differ significantly from the 50^th^ percentile for presepsin in neonatal blood previously reported in the literature [14].

## Conclusions

For the first time, we reported a cut-off of presepsin in the cord blood of term and preterm infants to predict clinical EOS. The use of biomarkers to decrease antibiotics administration in EOS should be one of the antibiotic stewardship targets in every neonatology unit [28]. Presepsin seems to be a promising candidate and our data could be the starting point to realize multicenter studies, confirming its feasibility in the management of antibiotic therapy in neonates with risk factors for EOS.

## Data Availability

The datasets used and/or analyzed during the current study are available from the corresponding author on reasonable request. All data generated or analyzed during this study are included in this published article.

## Abbreviations

AGA: Appropriate for Gestational Age
BC: Blood Culture
CRP: C-reactive protein
EOS: Ealy Onset Sepsis
ELBW: Extremely Low Birth Weight
GA: Gestational Age
GBS: Group B Streptococcus
IL- β: Interleukin-1β
IL-6: Interleukin-6
IL-8: Interleukin-8
LGA: Large for Gestational Age
NICU: Neonatal Intensive Care Unit
PCT: Procalcitonin
PROM: Premature Rupture of Membranes
PSEP: Presepsin
sCD14-ST: Soluble CD14 Subtype
SGA: Small for Gestational Age
TNF α: Tumor Necrosis Factor α
VLBW: Very Low Birth Weight

## References

[1] Simonsen KA, Anderson-Berry AL, Delair SF, Dele DH (2014). Early-onset neonatal sepsis. Clin Microbiol Rev.

[2] Hincu MA, Zonda GI, Stanciu GD, Nemescu D, Paduraru L (2020). Relevance of biomarkers currently in use or research for practical diagnosis approach of neonatal early-onset sepsis. Children.

[3] Hayes R, Hartnett J, Semova G, Murray C, Murphy K, Carroll L, et al. Neonatal sepsis definitions from randomised clinical trials. Pediatr Res 2021:0–8. 10.1038/s41390-021-01749-3. 10.1038/s41390-021-01749-3 PMC10132965 34743180

[4] Ray S, Sundaram V, Dutta S, Kumar P (2021). Ensuring administration of first dose of antibiotics within the golden hour of management in neonates with sepsis. BMJ Open Qual.

[5] Puopolo KM, Benitz WE, Zaoutis TE, Committee on Fetus and Newborn, Committee on Infectious Diseases (2018). Management of neonates born at >35 0/7 weeks’ gestation with suspected or proven early-onset bacterial sepsis. Pediatr.

[6] Chiesa C, Natale F, Pascone R, Osborn JF, Pacifico L, Bonci E (2011). C reactive protein and procalcitonin: Reference intervals for preterm and term newborns during the early neonatal period. Clin Chim Acta.

[7] de Rose DU, Perri A, Auriti C, Gallini F, Maggio L, Fiori B (2021). Time to positivity of blood cultures could inform decisions on antibiotics administration in neonatal early-onset sepsis. Antibiot.

[8] Maddaloni C, de Rose DU, Santisi A, Martini L, Caoci S, Bersani I (2021). The emerging role of presepsin (P-sep) in the diagnosis of sepsis in the critically ill infant: A literature review. Int J Mol Sci.

[9] Poggi C, Lucenteforte E, Petri D, de Masi S, Dani C (2022). Presepsin for the Diagnosis of Neonatal Early-Onset Sepsis: A Systematic Review and Meta-analysis. JAMA Pediatr.

[10] Seliem W, Sultan AM (2018). Presepsin as a predictor of early onset neonatal sepsis in the umbilical cord blood of premature infants with premature rupture of membranes. Pediatr Int.

[11] Verani JR, McGee L, Schrag SJ (2010). Prevention of Perinatal Group B Streptococcal Disease. MMWR Recommendations and Reports.

[12] Vermont Oxford Network. Manual of Operations: Part 2 - Data Definitions & Infant Data Forms 2019;Release 23:101.

[13] Achten NB, Klingenberg C, Benitz WE, Stocker M, Schlapbach LJ, Giannoni E (2019). Association of Use of the Neonatal Early-Onset Sepsis Calculator With Reduction in Antibiotic Therapy and Safety A Systematic Review and Meta-analysis. JAMA Pediatr.

[14] Pugni L, Pietrasanta C, Milani S, Vener C, Ronchi A, Falbo M (2015). Presepsin (soluble CD14 subtype): Reference ranges of a new sepsis marker in term and preterm neonates. PLoS ONE.

[15] Taneja R, Batra P (2021). Biomarkers as point of care tests (POCT) in neonatal sepsis: A state of science review. J Neonatal Perinatal Med.

[16] Abdel Motalib T, Khalaf FA, el Hendawy G (2015). Soluble CD14 - Subtype (Presepsin) and Hepcidin as Diagnostic and Prognostic Markers in Early Onset Neonatal Sepsis. Egypt J of Med Microbiol.

[17] Ozdemir AA, Elgormus Y (2017). Diagnostic Value of Presepsin in Detection of Early-Onset Neonatal Sepsis. Am J Perinatol.

[18] Montaldo P, Rosso R, Santantonio A, Chello G, Giliberti P (2017). Presepsin for the detection of early-onset sepsis in preterm newborns. Pediatr Res.

[19] Mussap M, Puxeddu E, Puddu M, Ottonello G, Coghe F, Comite P (2015). Soluble CD14 subtype (sCD14-ST) presepsin in premature and full term critically ill newborns with sepsis and SIRS. Clin Chim Acta.

[20] Pietrasanta C, Ronchi A, Vener C, Poggi C, Ballerini C, Testa L (2021). Presepsin (Soluble cd14 subtype) as an early marker of neonatal sepsis and septic shock: A prospective diagnostic trial. Antibiotics.

[21] Shane AL, Sánchez PJ, Stoll BJ (2017). Neonatal sepsis. The Lancet.

[22] Mukhopadhyay S, Sengupta S, Puopolo KM (2019). Challenges and opportunities for antibiotic stewardship among preterm infants. Arch Dis Child Fetal Neonatal Ed.

[23] Esaiassen E, Fjalstad JW, Juvet LK, van den Anker JN, Klingenberg C (2017). Antibiotic exposure in neonates and early adverse outcomes: a systematic review and meta-analysis. J Antimicrob Chemother.

[24] Stoll ABJ, Hansen NI, Watterberg KL, Bell EF, Michele C, Schibler K (2011). Early Onset Neonatal Sepsis: The Burden of Group B Streptococcal and E. coli Disease Continues. Pediatrics.

[25] Endo S, Suzuki Y, Takahashi G, Shozushima T, Ishikura H, Murai A (2012). Usefulness of presepsin in the diagnosis of sepsis in a multicenter prospective study. J Infect Chemother.

[26] Levy O (2007). Innate immunity of the newborn: Basic mechanisms and clinical correlates. Nat Rev Immunol.

[27] Fan Y, Yu JL (2012). Umbilical blood biomarkers for predicting early-onset neonatal sepsis. World Journal of Pediatrics.

[28] Katz S, Banerjee R, Schwenk H (2021). Antibiotic Stewardship for the Neonatologist and Perinatologist. Clin Perinatol.

